# Interleukin-6 Is a Risk Factor for Atrial Fibrillation in Chronic Kidney Disease: Findings from the CRIC Study

**DOI:** 10.1371/journal.pone.0148189

**Published:** 2016-02-03

**Authors:** Richard L. Amdur, Monica Mukherjee, Alan Go, Ian R. Barrows, Ali Ramezani, Jun Shoji, Muredach P. Reilly, Joseph Gnanaraj, Raj Deo, Sylvia Roas, Martin Keane, Steve Master, Valerie Teal, Elsayed Z. Soliman, Peter Yang, Harold Feldman, John W. Kusek, Cynthia M. Tracy, Dominic S. Raj, CRIC Study Investigators

**Affiliations:** 1 Biostatistics core, George Washington University Medical Faculty Associates, Washington, DC, United States of America; 2 Division of cardiology, Johns Hopkins University School of Medicine, Baltimore, MD, United States of America; 3 Kaiser Permanente Division of Research, Oakland, CA, United States of America; 4 George Washington University School of Medicine, Washington, DC, United States of America; 5 Division of Renal diseases and Hypertension, George Washington University School of Medicine, Washington, DC, United States of America; 6 Cardiovascular Institute, University of Pennsylvania, Philadelphia, PA, United States of America; 7 Bridgeport Hospital, Bridgeport, CT, United States of America; 8 Harvard Medical School, Joslin Diabetes Center, Beth Israel Deaconess Medical Center, Boston, MA, United States of America; 9 Temple Heart and Vascular Center, Philadelphia, PA, United States of America; 10 Pathology and Laboratory Medicine, Perelman School of Medicine at the University of Pennsylvania, Philadelphia, PA, United States of America; 11 Department of Biostatistics and Epidemiology, Perelman School of Medicine at the University of Pennsylvania, Philadelphia, PA, United States of America; 12 Wake Forest University School of Medicine, Winston-Salem, NC, United States of America; 13 National Institute of Diabetes and Digestive and Kidney Diseases, Division of Kidney, Urologic and Hematologic Diseases, Bethesda, MD, United States of America; 14 Division of Cardiology, George Washington University School of Medicine, Washington, DC, United States of America; Hospital Universitario de La Princesa, SPAIN

## Abstract

Atrial fibrillation (AF) is the most common sustained arrhythmia in patients with chronic kidney disease (CKD). In this study, we examined the association between inflammation and AF in 3,762 adults with CKD, enrolled in the Chronic Renal Insufficiency Cohort (CRIC) study. AF was determined at baseline by self-report and electrocardiogram (ECG). Plasma concentrations of interleukin(IL)-1, IL-1 Receptor antagonist, IL-6, tumor necrosis factor (TNF)-α, transforming growth factor-β, high sensitivity C-Reactive protein, and fibrinogen, measured at baseline. At baseline, 642 subjects had history of AF, but only 44 had AF in ECG recording. During a mean follow-up of 3.7 years, 108 subjects developed new-onset AF. There was no significant association between inflammatory biomarkers and past history of AF. After adjustment for demographic characteristics, comorbid conditions, laboratory values, echocardiographic variables, and medication use, plasma IL-6 level was significantly associated with presence of AF at baseline (Odds ratio [OR], 1.61; 95% confidence interval [CI], 1.21 to 2.14; *P* = 0.001) and new-onset AF (OR, 1.25; 95% CI, 1.02 to 1.53; *P* = 0.03). To summarize, plasma IL-6 level is an independent and consistent predictor of AF in patients with CKD.

## Introduction

Atrial fibrillation (AF) is the most common type of arrhythmia, affecting about 0.9% of the general population.[1,2] AF is associated with high health care system utilization, lower quality of life, and increased risk for hospitalization, heart failure, stroke and death.[3–5] Compared to the general population, the prevalence of AF is two to three times higher in those with chronic kidney disease (CKD).[6–8] In patients with CKD, incident AF is associated with an increase in mortality and progression to end-stage renal disease.[9,10] Therefore, risk assessment is of utmost importance in order to improve primary and secondary prevention of AF and its consequences in CKD patients.

Experimental as well as epidemiological studies suggest that inflammation may be involved in the pathogenesis of AF.[11] Inflammatory biomarkers, including C-reactive protein (CRP), interleukin (IL)-6, and tumor necrosis factor (TNF)-α are associated with the presence, persistence and outcome of AF in the general population.[12–14] CKD is a well-recognized pro-inflammatory state with elevated levels of pro-inflammatory cytokines and positive acute phase proteins.[15] However, no large scale studies have examined the association between AF and biomarkers of inflammation in CKD patients.

In this study, we examined the hypothesis that inflammation is associated with increased risk for AF in patients with CKD using the data from the Chronic Renal Insufficiency Cohort (CRIC) study. The CRIC study is an ongoing, multi-center, multi-ethnic, prospective observational cohort, established by the National Institute of Health with the goal of identifying the risk factors for cardiovascular disease (CVD) in patients with CKD.[16] Identifying the specific cytokine associated with AF may offer new opportunities for diagnosis, risk prediction and novel therapeutic strategies.

## Materials and Methods

### Study participants

The CRIC Study is an ongoing multicenter cohort study, in which 3,939 participants were enrolled from seven clinical centers in the US between June 2003 and August 2008. The organization, design, and methods of CRIC study have been previously reported.[17] The exclusion criteria in CRIC were cirrhosis, class III or IV heart failure, human immunodeficiency virus infection, cancer, autoimmune disease, polycystic kidney disease, pregnancy, post-transplant patients, current or past immunotherapy within the past six month or systemic chemotherapy. The study protocol was approved by the Institutional Review Boards at the participating clinical site which were Ann Arbor, Michigan; Baltimore, Maryland; Chicago, Illinois; Cleveland, Ohio; New Orleans, Louisiana; Philadelphia, Pennsylvania; and Oakland, California. Written informed consent was obtained from all study participants. (dbGaP Study Accession: phs000524.v1.p1)

### Data collection

Demographic and clinical characteristics were determined at baseline. Serum creatinine was measured by the Jaffe method on a Beckman Synchron System. Glomerular filtration rate was calculated using the estimating equation derived from the CRIC cohort (eGFR).[18] Albuminuria was estimated as the ratio of albumin to creatinine in the urine (UACR).

Diabetes was defined as fasting glucose ≥ 126 mg/d, random glucose ≥ 200 mg/dL, or use of insulin or anti-diabetic medication. Hypertension was defined as systolic blood pressure (SBP) ≥140 mm Hg, and/or diastolic blood pressure (DBP) ≥ 90 mm Hg, and/or self-reported antihypertensive medication use. Echocardiography (echo) was performed within 14 months of enrollment in the study according to the recommendations of the American Society of Echocardiography and interpreted at a central laboratory.[19] Left ventricular (LV) mass was calculated using the area–length method and indexed to height^2.7^ (LVMI). LV end-diastolic volume (LVEDV) and LV end-systolic volume (LVESV) were calculated using the modified biplane method. Ejection fraction was calculated as (LVEDV-LVESV)/LVEDV. Left atrium (LA) diameter was measured in the parasternal long axis view from trailing edge of the posterior aortic root-anterior LA complex to the posterior LA wall at end-systole.

### Measurement of biomarkers of inflammation

Methods to measure inflammatory biomarkers in CRIC study has been described previously.[15] All cytokine assays were performed in duplicate and mean values were used in the analysis. High sensitivity sandwich ELISAs (Quantikine HS, R&D Systems, Minneapolis, MN) were used to measure plasma IL-6, and TNF-α levels. The samples were stored at -80°C and assays performed at the time of initial thawing. The CV was <13% for all cytokines assays except for TNF-α and TGF-β, for which the estimated imprecision were 15.2% and 21.5%, respectively. High sensitivity (hs)-CRP and fibrinogen were quantified in EDTA plasma samples using specific laser-based immunonephelometric methods on the BNII (Siemens Healthcare Diagnostics, Deerfield, IL). The coefficient of variation for hs-CRP and fibrinogen were < 5%.

### Determination of AF

Standard 12-lead electrocardiographs (ECG) were recorded in all participants by strictly standardized procedures using identical ECG equipment (GE MAC 1200, GE Medical Systems, Milwaukee, WI). The digitally recorded ECGs were transmitted to a central ECG Reading Center. ECGs were analyzed using Minnesota Code for ECG classification.[20] A diagnosis of new-onset AF required that the participant developed first occurrence of AF during follow-up.

### Statistical Analysis

Histograms and Quantile-Quantile (QQ) Plots were examined, and skewed variables were log-transformed. Most biomarkers were severely skewed with outliers, even after log-transforming. Therefore, we examined the biomarkers as continuous variable and also as tertiles. Associations between patient characteristics and baseline AF were tested using chi-square for categorical variables and independent-groups t-tests for continuous variables. Logistic regression was used to examine the association of individual inflammatory biomarkers with outcome, adjusting for covariates. This was done in a phased approach, first testing a model that included patient demographic characteristics (age, sex, race, body mass index) and comorbid conditions (diabetes mellitus, smoking, history of CVD) in phase one, echocardiographic parameters and medications in phase two (SBP, eGFR, and use of Angiotensin converting enzyme inhibitor/Angiotensin receptor blocker [ACEi/ARB], antiplatelet agents, beta-blocker, calcium channel-blocker, diuretic, insulin, aspirin, and statins), and UACR and potassium in phase three. Predictors with *P*<0.10 from phases 1–3 were then used together as covariates to test each inflammatory marker in phase four. Interactions between cytokines in the final model and sex and race were also tested. Weighted regression was used to test for multicollinearity.[21] Tolerance and variance inflation factor were examined for each variable in the regression model.

For cytokines found to have significant association with new-onset AF after adjusting for covariates, time from baseline to incident AF was examined by tertile, and differences in time functions were tested with the log-rank test using Kaplan-Meier analysis. SAS (version 9.2, Cary, NC) was used for all data analysis, with *P*<0.05 indicating significance.

## Results

We could not determine current AF status for 177 patients who did not have a baseline ECG reading, and they were excluded from analyses. A total of 634 participants reported a past history of AF at baseline, but only 44 had AF by ECG. Table 1 shows the clinical characteristics of patients with and without AF at baseline based on either self-reported or ECG-diagnosed AF. Patients with AF were more likely to be older, African Americans, with history of CVD, larger body mass index (BMI) and lower eGFR. These subjects were more likely to be treated with beta blockers, antiplatelet agents and diuretics, and more likely to have lower ejection fraction, higher LVMI and larger LA diameter. Plasma levels of fibrinogen, IL-6 and TNF-α were significantly higher in patients with AF.

**Table 1:** Baseline characteristics of patients with and without atrial fibrillation*.

Variable	AF, n = 642	No AF, n = 3,120	*P*-value
Age (years)	60.8 ± 9.4	57.0 ± 11.2	<0.001
Female (%)	296 (46.1%)	1398 (44.8%)	0.55
African American (%)	316 (49.2%)	1253 (40.2%)	<0.001
Caucasian (%)	270 (42.1%)	1479 (47.4%)	0.013
Hispanic (%)	49 (7.6%)	436 (14.0%)	<0.001
Hypertension (%)	563 (87.7%)	2674 (85.7%)	0.19
Diabetes mellitus (%)	332 (51.7%)	1493 (47.9%)	0.08
History of CHF (%)	177 (27.6%)	189 (6.1%)	<0.001
History of CVD (%)	377 (58.7%)	883 (28.3%)	<0.001
BMI(kg/m^2^)	32.8 ± 8.2	31.9 ± 7.7	0.005
eGFR (ml/min/1.73 m^2^)	41.6 ± 13.2	43.1 ± 13.6	0.02
Potassium (mmol/L)	4.3 ± (0.5)	4.4 ± (0.5)	0.04
UACR ug/mg (median, IQR)	44.0 (9.3–367.8)	53.1 (8.3–479.3)	0.12
Log-BNP	4.3 ± 1.4	3.6 ± 1.3	<0.0001
**Medication use (%)**			
ACEi/ARB	456 (71.5%)	2112 (68.2%)	0.10
Antiplatelet agents	335 (52.5%)	1393 (45.0%)	<0.001
Beta-blocker	422 (66.1%)	1428 (46.1%)	<0.001
Calcium Channel Blocker	277 (43.4%)	1238 (40.0%)	0.11
Diuretic	461 (72.3%)	1767 (57.0%)	<0.001
Aspirin	310 (48.6%)	1301 (42.0%)	0.002
**Echocardiographic data**			
EF (%)	51.4 ± 11.4	54.5 ± 7.9	<0.001
LVMI	74.6 ± 27.5	64.2 ± 23.1	<0.001
LA diameter (cm)	4.1 ± 0.7	3.9 ± 0.6	<0.001
**Biomarkers**			
hs-CRP (mg/L)	6.0 ± 9.2	5.5 ± 9.9	0.16
Fibrinogen (g/L)	4.3 ± 1.1	4.1 ± 1.2	<0.001
IL-1β (pg/ml)	1.4 ± 4.2	1.4 ± 4.8	0.9
IL-1RA (pg/ml)	1475.6 ± 2685.0	1421.8 ± 1910.1	0.63
IL-6 (pg/ml)	4.6 ± 13.8	3.4 ± 9.1	0.0079
TNF-α (pg/ml)	2.9 ± 2.7	3.5 ± 13.9	0.02
TGF-β (pg/ml)	13.5 ± 10.8	13.9 ± 11.2	0.50

*AF was defined as having either self-reported or ECG-diagnosed AF.

Data presented as n (%) or mean ± sd. CVD = Cardiovascular disease, CHF = Congestive heart failure, BMI = Body mass index, eGFR = Estimated glomerular filtration rate, UACR = Urine albumin to creatinine ratio, ACEi/ARB = Angiotensin converting enzyme inhibitor/Angiotensin receptor blocker, CCB = Calcium channel blockers, EF = Ejection fraction, LVMI = Left ventricular mass index, LA = Left atrium, interleukin = IL, IL-1RA = IL-1 Receptor antagonist, TNF-α = tumor necrosis factor-α, TGF-β = Transforming growth factor-β, hs-CRP = high sensitivity C-Reactive protein

### Atrial fibrillation at baseline

**Fig 1:**
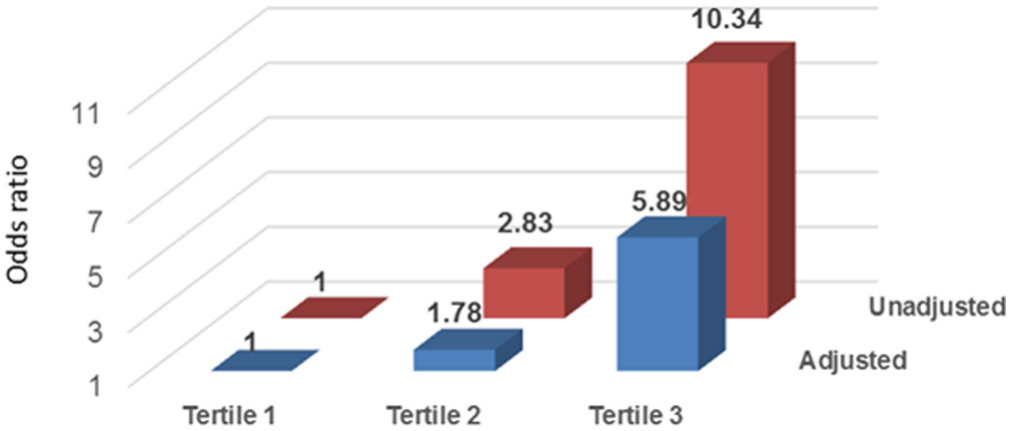
Unadjusted and multivariable adjusted association between tertiles of IL-6 and ECG-diagnosed atrial fibrillation at baseline.

Among the biomarkers studied, highest tertiles of hs-CRP (odds ratio [OR], 1.47; 95% confidence interval [CI], 1.19 to 1.81; *P*<0.001) and IL-6 (OR, 1.87; 95% CI, 1.50 to 2.32; *P*<0.001) had significant univariate associations with past history of AF. However, these associations were attenuated after adjustment for demographic characteristics, comorbid conditions, echo parameters, and medications use, and were no longer significant. Among the biomarkers tested, only IL-6 was significantly associated with AF confirmed by ECG in the fully adjusted model. (OR, 1.61; 95% CI, 1.21 to 2.14; *P* = 0.001). (Table 2) The odds for presence of AF by ECG at baseline was six fold higher for the third tertile of IL-6 compared to the lowest tertile (OR, 5.89; 95% CI, 1.75 to 19.81; *P* = 0.004). (Fig 1)

**Table 2:** Association between inflammatory biomarkers and atrial fibrillation diagnosed by ECG.

	Unadjusted OR	*P*-value	Adjusted OR†	*P*-value
**ECG diagnosed AF at baseline**				
hs-CRP	1.15 (0.91–1.46)	0.24	1.12 (0.86–1.45)	0.40
Fibrinogen	1.12 (0.89–1.42)	0.33	1.06 (0.82–1.37)	0.66
TNF-α	1.22 (0.83–1.81)	0.31	1.01 (0.64–1.60)	0.9
IL-6	1.70 (1.36–2.12)	<0.0001	1.61 (1.21–2.14)	0.001
**New-onset atrial fibrillation**				
hs-CRP	1.16 (1.00–1.35)	0.049	1.13 (0.96–1.33)	0.13
Fibrinogen	1.09 (0.93–1.27)	0.28	1.01 (0.86–1.18)	0.9
TNF-α	1.15 (0.89–1.48)	0.29	0.97 (0.73–1.28)	0.82
IL-6	1.37 (1.16–1.63)	0.0007	1.25 (1.02–1.53)	0.03

^†^ Adjusted for age, sex, race, body mass index, diabetes mellitus, smoking, history of CVD, echocardiographic parameters, SBP, eGFR, and use of Angiotensin converting enzyme inhibitor/Angiotensin receptor blocker [ACEi/ARB], antiplatelet agents, beta-blocker, calcium channel-blocker, diuretic, insulin, aspirin, and statins, and UACR and potassium.

### New-onset atrial fibrillation

**Fig 2:**
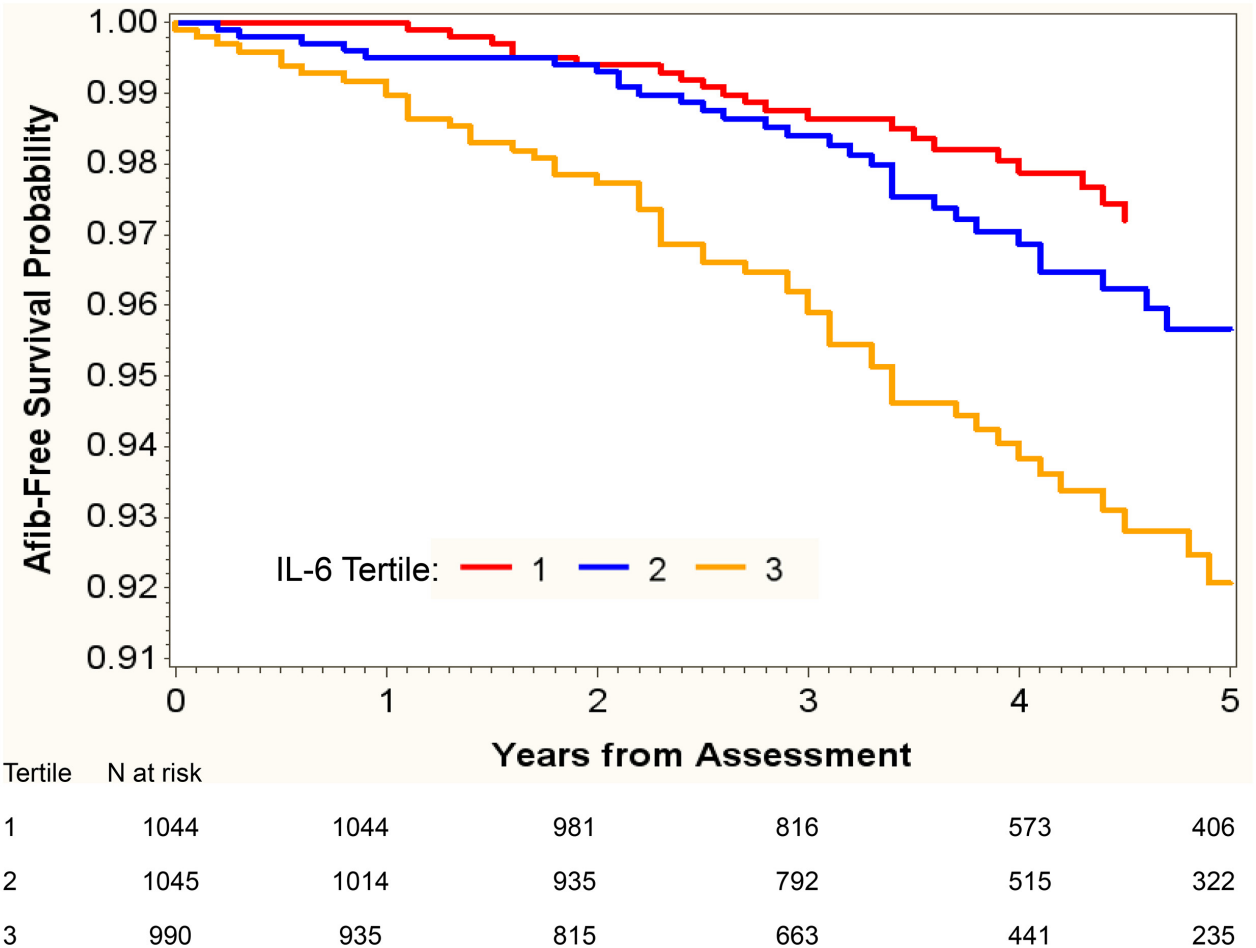
Kaplan-Meier incident atrial fibrillation-free survival estimates by IL-6 tertile. Kaplan Meier AF-free survival estimates for new-onset AF (in patients who were negative for history of AF or current AF) stratified by IL-6 tertiles. Time to AF differs significantly between tertiles (Log-Rank chi-square = 24.16, df = 2, *P*<0.001).

**Fig 3:**
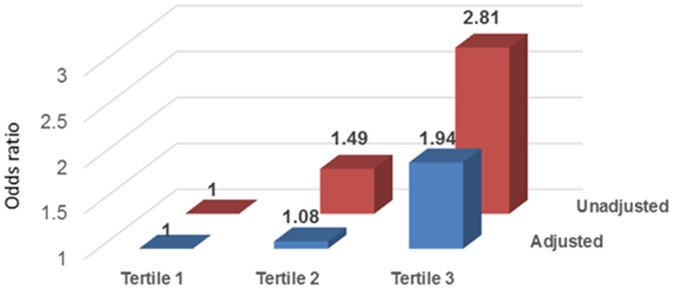
Unadjusted and multivariable adjusted association between tertiles of IL-6 and new-onset atrial fibrillation during follow-up.

During a mean follow-up of 3.7 (interquartile range 2.8 to 4.9) years, 108 subjects developed new-onset AF. Kaplan-Meier (KM) analysis was used to examine time from baseline assessment to incident AF for tertiles of IL-6. The time functions for IL-6 tertiles were significantly different (*P*<0.001; Fig 2). Patients with the highest IL-6 tertile were most likely to have new-onset of AF during follow-up. In the unadjusted model, hs-CRP (OR, 1.16; 95% CI, 1.00 to 1.35; *P* = 0.05) and IL-6 (OR, 1.37; 95% CI, 1.16 to 1.63; *P*<0.001) were associated with new onset AF. After adjusting for demographic characteristics, comorbid conditions, laboratory values, echocardiographic variables, and medication use, only IL-6 was associated with new-onset AF (OR, 1.25; 95% CI, 1.02 to 1.53; *P* = 0.03). (Table 2) As compared to the lowest tertile, the highest IL-6 tertile was associated with two-fold increased risk for new-onset AF (OR, 1.94; 95% CI, 1.14 to 3.31; *P* = 0.02). (Fig 3)

Interactions of cytokine levels with sex and race were not significant. Weighted regression was used to test for multicollinearity in prediction models for incident AF.[21] In these models the largest variance inflation was <1.3, indicating that multicollinearity was not distorting the parameter p-values.

## Discussion

We examined the association of selected biomarkers of inflammation with AF in a large cohort of CKD subjects with a wide range of kidney function. Among the biomarkers tested, only IL-6 emerged as a strong independent risk factor for AF at baseline visit and new AF. Compared to those in the lowest tertile, patients in the highest tertile of IL-6 had about 6-fold higher risk for having AF by ECG at baseline and 2-fold higher risk for new-onset AF during follow-up. The association between AF and IL-6 remained significant after adjusting for age, sex, race, BMI, smoking, diabetes mellitus, history of CVD, laboratory variables, SBP, echo parameters, medication use and kidney function.

CKD is a well-recognized risk factor for incident as well as prevalent AF.[6,9] Among patients with coronary artery disease, decreased cystatin C–based eGFR and albuminuria are associated with prevalent AF.[22] In Cardiovascular Health Study participant’s, cystatin C was associated with prevalent AF, but neither cystatin C nor eGFR were independent predictors of incident AF.[23] AF has been associated with older age, smoking, male sex and history of CVD, but not with eGFR among the CRIC study participants.[24] However, a substantial proportion of AF risk cannot be explained by traditional risk factors alone. Other factors such as inflammation, abnormal cardiac geometry, activation of renin-angiotensin-aldosterone system and sympathetic over activity could also be potential cause for increased prevalence of AF in CKD.[25–27] In the present study, we found elevated level of plasma IL-6 to be an independent and consistent risk factor for AF in CKD patients.

There is an increasing body of evidence linking inflammation to AF. Cytokines are secreted polypeptides that regulate the inflammatory response through autocrine, paracrine and endocrine mechanisms.[28,29] These biomolecules are pleiotropic in their actions, with considerable redundancy between their functions.[30] Cytokines may mediate downstream effects through acute phase proteins such as C-Reactive Protein (CRP) and fibrinogen. Atrial tissue in patients with AF shows evidence of inflammatory infiltrate.[31] TNF-α induces abnormal calcium signaling and augments arrythmogenicity in isolated rabbit pulmonary vein cardiomyocytes.[32] Findings from Women’s Health Study showed that an inflammation score comprised of plasma levels of hs-CRP, soluble intercellular adhesion molecule 1 (sICAM-1), and fibrinogen is associated with the risk of incident AF.[33] In Cardiovascular Health Study, a population-based, longitudinal study of coronary heart disease and stroke in adults aged 65 years and older, CRP was independently associated with baseline as well as future development of AF.[34] Analysis of ten biomarkers indicative of pathways related to AF in the Framingham cohort participants identified brain natriuretic peptide as the strongest predictor of incident AF.[35] CRP was also significantly associated with the outcome, but it did not improve risk prediction further.[35] A retrospective cross-sectional study in 1,010 CKD patients did not note any association between hs-CRP levels and presence of AF.[36] This is consistent with our finding in this study.

In multivariate analysis plasma IL-6 is associated with AF at baseline as well as new-onset AF during follow-up. IL-6 is a pleiotropic cytokine with diverse biological function.[37] It is produced not only by immune cells, but also by endothelial cells, vascular smooth-muscle cells, and ischemic myocytes. We recently reported that elevated plasma levels of hs-CRP and IL-6 are associated with left ventricular hypertrophy and systolic dysfunction in CRIC study participants.[27] IL-6 has been linked to the initiation and perpetuation of AF in the setting of coronary heart disease, post-coronary artery bypass grafting, post-cardioversion and after radiofrequency catheter ablation.[13,38–40] Circulating level of IL-6 is associated with activation of sympathetic and renin-angiotensin systems as well as LA diameter and severity of LV dysfunction, which are established risk factors for AF.[41,42] Furthermore, IL-6 may augment the risk for thromboembolism through increase in expression of fibrinogen, tissue factor, factor VIII and von Willebrand factor.[43] Plasma level of CRP, IL-6, fibrinogen, TNF-α, CD-40 ligand and monocyte chemoattractant protein-1 were measured in 971 Heart and Soul Study, which consisted of older adults with stable coronary heart disease.[13] However, after controlling for comorbidities, only IL-6 was significantly associated with AF.[13]

Elevated plasma level of IL-6 has been found to correlate with increased mortality in general population, elderly and in patients with CKD.[44–47] Barreto et al noted that IL-6 is a significantly better predictor of all cause and cardiovascular mortality than CRP, albumin and TNF-α in patients with CKD.[48] Zocalli showed that an inflammation score composed of CRP, IL-6, IL-1, IL-18, and TNF-α predicts death no better than IL-6 alone in ESRD patients.[49] CRP is elevated in patients with AF and is also a predictive of new onset AF in Cardiovascular Health Study participants.[34]

We observed a strong association between IL-6 and AF, but not with CRP in CRIC study participants. CRP is an acute phase protein synthesized primarily by the liver, which is transcriptionally regulated by IL-6. The precise mechanism by which IL-6 and CRP induces AF is uncertain, but might reflect active participation in atrial remodeling. Indeed, circulating IL-6 level is associated with increased left atrial size, supporting a link between the cytokine and atrial remodeling.[41] In Multi-Ethnic Study of Atherosclerosis (MESA) study population, elevated IL-6 was associated with depressed LV systolic function, which was shown to be independent of known cardiovascular risk factors and CRP.[50] These finding argue that IL-6 may be a better predictor of the inflammatory effects in the myocardium in the general population as well as in patients with CKD.

IL-6 signaling directed interventions are most logical strategies for reducing inflammation. IL-6 first binds to a non-signaling IL-6 Receptor (IL-6R), which, after dimerization with gp130, leads to activation of receptor-associated kinases within the cell. Interventions directed against IL-6/gp130 signaling are potential targets for therapy. Besides the humanized IL-6R antibody tocilizumab, a number of anti–IL-6 molecules are in pipeline.[51] The utility of such therapies in inflammation associated with CKD remains to be explored.

Our study has a number of strengths, which include (a) a large multiracial cohort of patients with a broad range of kidney function, (b) detailed and comprehensive data collection, (c) longitudinal data with a mean follow up of 3.7 years, and (d) examination of a large panel of biomarkers of inflammation. These findings should be considered within the context of some limitations: Cytokines were measured only at baseline in our study. However, investigators have shown that even a single baseline measure could accurately reflect healthy individuals' inflammatory status over a four to six month period.[52] Although our findings support the potential role of inflammation in AF, causality remains to be established.

To summarize, we examined selected inflammatory biomarkers as risk factors for AF in CRIC study participants and found that only elevated levels of IL-6 is associated with increased risk for ECG-diagnosed AF at baseline and also new-onset AF during follow-up. Elevated IL-6 may be useful in risk stratification and also as a potential therapeutic target in the management of high risk CKD patients.

## References

[1] Go AS, Hylek EM, Phillips KA, Chang Y, Henault LE, Selby JV et al (2001) Prevalence of diagnosed atrial fibrillation in adults: national implications for rhythm management and stroke prevention: the AnTicoagulation and Risk Factors in Atrial Fibrillation (ATRIA) Study. JAMA 285: 2370–2375. jcc10004 [pii]. 11343485 10.1001/jama.285.18.2370

[2] Fuster V, Ryden LE, Asinger RW, Cannom DS, Crijns HJ, Frye RL et al (2001) ACC/AHA/ESC Guidelines for the Management of Patients With Atrial Fibrillation: Executive Summary A Report of the American College of Cardiology/American Heart Association Task Force on Practice Guidelines and the European Society of Cardiology Committee for Practice Guidelines and Policy Conferences (Committee to Develop Guidelines for the Management of Patients With Atrial Fibrillation) Developed in Collaboration With the North American Society of Pacing and Electrophysiology. Circulation 104: 2118–2150. 11673357

[3] Wolf PA, Abbott RD, Kannel WB (1991) Atrial fibrillation as an independent risk factor for stroke: the Framingham Study. Stroke 22: 983–988. 1866765 10.1161/01.str.22.8.983

[4] Benjamin EJ, Wolf PA, D'Agostino RB, Silbershatz H, Kannel WB, Levy D (1998) Impact of atrial fibrillation on the risk of death: the Framingham Heart Study. Circulation 98: 946–952. 9737513 10.1161/01.cir.98.10.946

[5] Miyasaka Y, Barnes ME, Gersh BJ, Cha SS, Bailey KR, Seward JB et al (2008) Changing trends of hospital utilization in patients after their first episode of atrial fibrillation. Am J Cardiol 102: 568–572. S0002-9149(08)00749-2 [pii]; 10.1016/j.amjcard.2008.04.025 18721513 PMC3743254

[6] Baber U, Howard VJ, Halperin JL, Soliman EZ, Zhang X, McClellan W et al (2011) Association of chronic kidney disease with atrial fibrillation among adults in the United States: REasons for Geographic and Racial Differences in Stroke (REGARDS) Study. Circ Arrhythm Electrophysiol 4: 26–32. CIRCEP.110.957100 [pii]; 10.1161/CIRCEP.110.957100 21076159 PMC3049935

[7] Genovesi S, Pogliani D, Faini A, Valsecchi MG, Riva A, Stefani F et al (2005) Prevalence of atrial fibrillation and associated factors in a population of long-term hemodialysis patients. Am J Kidney Dis 46: 897–902. S0272-6386(05)01055-3 [pii]; 10.1053/j.ajkd.2005.07.044 16253730

[8] Alonso A, Lopez FL, Matsushita K, Loehr LR, Agarwal SK, Chen LY et al (2011) Chronic kidney disease is associated with the incidence of atrial fibrillation: the Atherosclerosis Risk in Communities (ARIC) study. Circulation 123: 2946–2953. CIRCULATIONAHA.111.020982 [pii]; 10.1161/CIRCULATIONAHA.111.020982 21646496 PMC3139978

[9] Nelson SE, Shroff GR, Li S, Herzog CA (2012) Impact of chronic kidney disease on risk of incident atrial fibrillation and subsequent survival in medicare patients. J Am Heart Assoc 1: e002097 10.1161/JAHA.112.002097 jah371 [pii]. 23130165 PMC3487349

[10] Bansal N, Fan D, Hsu CY, Ordonez JD, Marcus GM, Go AS (2013) Incident atrial fibrillation and risk of end-stage renal disease in adults with chronic kidney disease. Circulation 127: 569–574. CIRCULATIONAHA.112.123992 [pii]; 10.1161/CIRCULATIONAHA.112.123992 23275377 PMC3676734

[11] Boos CJ, Anderson RA, Lip GY (2006) Is atrial fibrillation an inflammatory disorder? Eur Heart J 27: 136–149. ehi645 [pii]; 10.1093/eurheartj/ehi645 16278230

[12] Marott SC, Nordestgaard BG, Zacho J, Friberg J, Jensen GB, Tybjaerg-Hansen A et al (2010) Does elevated C-reactive protein increase atrial fibrillation risk? A Mendelian randomization of 47,000 individuals from the general population. J Am Coll Cardiol 56: 789–795. S0735-1097(10)02305-3 [pii]; 10.1016/j.jacc.2010.02.066 20797493

[13] Marcus GM, Whooley MA, Glidden DV, Pawlikowska L, Zaroff JG, Olgin JE (2008) Interleukin-6 and atrial fibrillation in patients with coronary artery disease: data from the Heart and Soul Study. Am Heart J 155: 303–309. S0002-8703(07)00764-8 [pii]; 10.1016/j.ahj.2007.09.006 18215601 PMC2247366

[14] Li J, Solus J, Chen Q, Rho YH, Milne G, Stein CM et al (2010) Role of inflammation and oxidative stress in atrial fibrillation. Heart Rhythm 7: 438–444. S1547-5271(09)01380-0 [pii]; 10.1016/j.hrthm.2009.12.009 20153266 PMC2843774

[15] Gupta J, Mitra N, Kanetsky PA, Devaney J, Wing MR, Reilly M et al (2012) Association between albuminuria, kidney function, and inflammatory biomarker profile. Clin J Am Soc Nephrol 7: 1938–1946. CJN.03500412 [pii]; 10.2215/CJN.03500412 23024164 PMC3513744

[16] Feldman HI, Appel LJ, Chertow GM, Cifelli D, Cizman B, Daugirdas J et al (2003) The Chronic Renal Insufficiency Cohort (CRIC) Study: Design and Methods. J Am Soc Nephrol 14: S148–S153. 12819321 10.1097/01.asn.0000070149.78399.ce

[17] Lash JP, Go AS, Appel LJ, He J, Ojo A, Rahman M et al (2009) Chronic Renal Insufficiency Cohort (CRIC) Study: baseline characteristics and associations with kidney function. Clin J Am Soc Nephrol 4: 1302–1311. CJN.00070109 [pii]; 10.2215/CJN.00070109 19541818 PMC2723966

[18] Anderson AH, Yang W, Hsu CY, Joffe MM, Leonard MB, Xie D et al (2012) Estimating GFR Among Participants in the Chronic Renal Insufficiency Cohort (CRIC) Study. Am J Kidney Dis 60: 250–261. S0272-6386(12)00674-9 [pii]; 10.1053/j.ajkd.2012.04.012 22658574 PMC3565578

[19] Lang RM, Bierig M, Devereux RB, Flachskampf FA, Foster E, Pellikka PA et al (2005) Recommendations for chamber quantification: a report from the American Society of Echocardiography's Guidelines and Standards Committee and the Chamber Quantification Writing Group, developed in conjunction with the European Association of Echocardiography, a branch of the European Society of Cardiology. J Am Soc Echocardiogr 18: 1440–1463. S0894-7317(05)00983-1 [pii]; 10.1016/j.echo.2005.10.005 16376782

[20] Prineas RJ, Crow RS, Blackburn H (1982) The Minnesota Code Manual of Electrocardiographic Findings.

[21] Allison PD (1999) Logistic regression using SAS: Theory and application.

[22] McManus DD, Corteville DC, Shlipak MG, Whooley MA, Ix JH (2009) Relation of kidney function and albuminuria with atrial fibrillation (from the Heart and Soul Study). Am J Cardiol 104: 1551–1555. S0002-9149(09)01392-7 [pii]; 10.1016/j.amjcard.2009.07.026 19932791 PMC2796571

[23] Deo R, Katz R, Kestenbaum B, Fried L, Sarnak MJ, Psaty BM et al (2010) Impaired kidney function and atrial fibrillation in elderly subjects. J Card Fail 16: 55–60. S1071-9164(09)00653-8 [pii]; 10.1016/j.cardfail.2009.07.002 20123319 PMC2818049

[24] Soliman EZ, Prineas RJ, Go AS, Xie D, Lash JP, Rahman M et al (2010) Chronic kidney disease and prevalent atrial fibrillation: the Chronic Renal Insufficiency Cohort (CRIC). Am Heart J 159: 1102–1107. S0002-8703(10)00253-X [pii]; 10.1016/j.ahj.2010.03.027 20569726 PMC2891979

[25] Ehrlich JR, Hohnloser SH, Nattel S (2006) Role of angiotensin system and effects of its inhibition in atrial fibrillation: clinical and experimental evidence. Eur Heart J 27: 512–518. ehi668 [pii]; 10.1093/eurheartj/ehi668 16311236

[26] Schlaich MP, Socratous F, Hennebry S, Eikelis N, Lambert EA, Straznicky N et al (2009) Sympathetic activation in chronic renal failure. J Am Soc Nephrol 20: 933–939. ASN.2008040402 [pii]; 10.1681/ASN.2008040402 18799718

[27] Gupta J, Dominic EA, Fink JC, Ojo AO, Barrows IR, Reilly MP et al (2015) Association between Inflammation and Cardiac Geometry in Chronic Kidney Disease: Findings from the CRIC Study. PLoS One 10: e0124772 10.1371/journal.pone.0124772 PONE-D-14-55447 [pii]. 25909952 PMC4409366

[28] Wing MR, Yang W, Teal V, Navaneethan S, Tao K, Ojo A et al (2014) Race modifies the association between adiposity and inflammation in patients with chronic kidney disease: Findings from the chronic renal insufficiency cohort study. Obesity (Silver Spring). 10.1002/oby.20692 PMC4327849 24415732

[29] Raj DS, Dominic EA, Pai A, Osman F, Morgan M, Pickett G et al (2005) Skeletal muscle, cytokines, and oxidative stress in end-stage renal disease. Kidney Int 68: 2338–2344. KID695 [pii]; 10.1111/j.1523-1755.2005.00695.x 16221238

[30] Feghali CA, Wright TM (1997) Cytokines in acute and chronic inflammation. Front Biosci 2: d12–d26. 9159205 10.2741/a171

[31] Frustaci A, Chimenti C, Bellocci F, Morgante E, Russo MA, Maseri A (1997) Histological substrate of atrial biopsies in patients with lone atrial fibrillation. Circulation 96: 1180–1184. 9286947 10.1161/01.cir.96.4.1180

[32] Lee SH, Chen YC, Chen YJ, Chang SL, Tai CT, Wongcharoen W et al (2007) Tumor necrosis factor-alpha alters calcium handling and increases arrhythmogenesis of pulmonary vein cardiomyocytes. Life Sci 80: 1806–1815. S0024-3205(07)00195-6 [pii]; 10.1016/j.lfs.2007.02.029 17383682

[33] Conen D, Ridker PM, Everett BM, Tedrow UB, Rose L, Cook NR et al (2010) A multimarker approach to assess the influence of inflammation on the incidence of atrial fibrillation in women. Eur Heart J 31: 1730–1736. ehq146 [pii]; 10.1093/eurheartj/ehq146 20501475 PMC2903714

[34] Aviles RJ, Martin DO, Apperson-Hansen C, Houghtaling PL, Rautaharju P, Kronmal RA et al (2003) Inflammation as a risk factor for atrial fibrillation. Circulation 108: 3006–3010. 10.1161/01.CIR.0000103131.70301.4F 01.CIR.0000103131.70301.4F [pii]. 14623805

[35] Schnabel RB, Larson MG, Yamamoto JF, Sullivan LM, Pencina MJ, Meigs JB et al (2010) Relations of biomarkers of distinct pathophysiological pathways and atrial fibrillation incidence in the community. Circulation 121: 200–207. CIRCULATIONAHA.109.882241 [pii]; 10.1161/CIRCULATIONAHA.109.882241 20048208 PMC3224826

[36] Ananthapanyasut W, Napan S, Rudolph EH, Harindhanavudhi T, Ayash H, Guglielmi KE et al (2010) Prevalence of atrial fibrillation and its predictors in nondialysis patients with chronic kidney disease. Clin J Am Soc Nephrol 5: 173–181. CJN.03170509 [pii]; 10.2215/CJN.03170509 20007681 PMC2827597

[37] Raj DS, Moseley P, Dominic EA, Onime A, Tzamaloukas AH, Boyd A et al (2008) Interleukin-6 modulates hepatic and muscle protein synthesis during hemodialysis. Kidney Int 73: 1054–1061. ki200821 [pii]; 10.1038/ki.2008.21 18288103

[38] Kaireviciute D, Blann AD, Balakrishnan B, Lane DA, Patel JV, Uzdavinys G et al (2010) Characterisation and validity of inflammatory biomarkers in the prediction of post-operative atrial fibrillation in coronary artery disease patients. Thromb Haemost 104: 122–127. 09-12-0837 [pii]; 10.1160/TH09-12-0837 20458440

[39] Leftheriotis DI, Fountoulaki KT, Flevari PG, Parissis JT, Panou FK, Andreadou IT et al (2009) The predictive value of inflammatory and oxidative markers following the successful cardioversion of persistent lone atrial fibrillation. Int J Cardiol 135: 361–369. S0167-5273(08)00563-9 [pii]; 10.1016/j.ijcard.2008.04.012 18640731

[40] Henningsen KM, Nilsson B, Bruunsgaard H, Chen X, Pedersen BK, Svendsen JH (2009) Prognostic impact of hs-CRP and IL-6 in patients undergoing radiofrequency catheter ablation for atrial fibrillation. Scand Cardiovasc J 43: 285–291. 907307162 [pii]; 10.1080/14017430802653676 19117239

[41] Psychari SN, Apostolou TS, Sinos L, Hamodraka E, Liakos G, Kremastinos DT (2005) Relation of elevated C-reactive protein and interleukin-6 levels to left atrial size and duration of episodes in patients with atrial fibrillation. Am J Cardiol 95: 764–767. S0002-9149(04)01885-5 [pii]; 10.1016/j.amjcard.2004.11.032 15757607

[42] Raymond RJ, Dehmer GJ, Theoharides TC, Deliargyris EN (2001) Elevated interleukin-6 levels in patients with asymptomatic left ventricular systolic dysfunction. Am Heart J 141: 435–438. S0002-8703(01)95658-3 [pii]; 10.1067/mhj.2001.113078 11231442

[43] Kerr R, Stirling D, Ludlam CA (2001) Interleukin 6 and haemostasis. Br J Haematol 115: 3–12. 3061 [pii]. 11722403 10.1046/j.1365-2141.2001.03061.x

[44] Ridker PM, Rifai N, Stampfer MJ, Hennekens CH (2000) Plasma concentration of interleukin-6 and the risk of future myocardial infarction among apparently healthy men. Circulation 101: 1767–1772. 10769275 10.1161/01.cir.101.15.1767

[45] Harris TB, Ferrucci L, Tracy RP, Corti MC, Wacholder S, Ettinger WH Jr.. et al (1999) Associations of elevated interleukin-6 and C-reactive protein levels with mortality in the elderly. Am J Med 106: 506–512. S0002934399000662 [pii]. 10335721 10.1016/s0002-9343(99)00066-2

[46] Kimmel PL, Phillips TM, Simmens SJ, Peterson RA, Weihs KL, Alleyne S et al (1998) Immunologic function and survival in hemodialysis patients. Kidney International 54: 236–244. 9648084 10.1046/j.1523-1755.1998.00981.x PMC6146918

[47] Rao M, Guo D, Perianayagam MC, Tighiouart H, Jaber BL, Pereira BJ et al (2005) Plasma interleukin-6 predicts cardiovascular mortality in hemodialysis patients. Am J Kidney Dis 45: 324–333. 15685511 10.1053/j.ajkd.2004.09.018

[48] Barreto DV, Barreto FC, Liabeuf S, Temmar M, Lemke HD, Tribouilloy C et al (2010) Plasma interleukin-6 is independently associated with mortality in both hemodialysis and pre-dialysis patients with chronic kidney disease. Kidney Int 77: 550–556. ki2009503 [pii]; 10.1038/ki.2009.503 20016471

[49] Zoccali C, Tripepi G, Mallamaci F (2006) Dissecting inflammation in ESRD: do cytokines and C-reactive protein have a complementary prognostic value for mortality in dialysis patients? J Am Soc Nephrol 17: S169–S173. 17/12_suppl_3/S169 [pii]; 10.1681/ASN.2006080910 17130257

[50] Yan AT, Yan RT, Cushman M, Redheuil A, Tracy RP, Arnett DK et al (2010) Relationship of interleukin-6 with regional and global left-ventricular function in asymptomatic individuals without clinical cardiovascular disease: insights from the Multi-Ethnic Study of Atherosclerosis. Eur Heart J 31: 875–882. ehp454 [pii]; 10.1093/eurheartj/ehp454 20064818 PMC2848322

[51] Jones SA, Scheller J, Rose-John S (2011) Therapeutic strategies for the clinical blockade of IL-6/gp130 signaling. J Clin Invest 121: 3375–3383. 57158 [pii]; 10.1172/JCI57158 21881215 PMC3163962

[52] Navarro SL, Brasky TM, Schwarz Y, Song X, Wang CY, Kristal AR et al (2012) Reliability of serum biomarkers of inflammation from repeated measures in healthy individuals. Cancer Epidemiol Biomarkers Prev 21: 1167–1170. 1055-9965.EPI-12-0110 [pii]; 10.1158/1055-9965.EPI-12-0110 22564866 PMC3392358

